# Prospect of Metal Ceramic (Titanium-Wollastonite) Composite as Permanent Bone Implants: A Narrative Review

**DOI:** 10.3390/ma14020277

**Published:** 2021-01-07

**Authors:** Lohashenpahan Shanmuganantha, Azmi Baharudin, Abu Bakar Sulong, Roslinda Shamsudin, Min Hwei Ng

**Affiliations:** 1 Department of Tissue Engineering, National University of Malaysia, Selangor Darul Ehsan 56000, Malaysia; shenpa_1991@outlook.com; 2 Department of Orthopaedic and Traumatology, National University of Malaysia, Selangor Darul Ehsan 56000, Malaysia; azmibaha@ppukm.ukm.edu.my; 3 Department of Mechanical Engineering, National University of Malaysia, Selangor Darul Ehsan 43600, Malaysia; abubakar@ukm.edu.my; 4 Department of Science and Technology, National University of Malaysia, Selangor Darul Ehsan 43600, Malaysia; azmi@ukm.edu.my

**Keywords:** titanium, wollastonite, bioceramic, mesenchymal stem cells bone implants

## Abstract

This literature review discusses the influence of titanium ceramic composites as a biomaterial towards the fabrication of implants for orthopedic applications. The concept of applying metal-ceramic composites enable many novel combinations in the design and fabrication of complex materials which enhances functionality to improve cell and tissue matrix interactions particularly in the formation of bone. Specific focus is placed on its plethora of materials selected from the metals and ceramic group and identifying the optimal combination that matches them. The prospect of wollastonite as the ceramic counterpart is also highlighted. In this review, we have highlighted the different fabrication methods for such metal-ceramic materials as well as the role that these hybrids play in an in vitro and in vivo environment. Its economic potential as a bone implant material is also discussed.

## 1. Introduction

The skeletal system contains an assortment of bones and joints in the body. Every bone is a complex living organ that is made up of different types of cells, protein fibers, and minerals. The skeleton acts as a scaffold by providing support and protection for the soft tissues that make up the rest of the body. Furthermore, the skeletal system also provides attachment points for muscles to allow movements at the joints. New blood cells are produced by the red bone marrow inside of our bones. Bones also act as the body’s warehouse for calcium, iron, and energy in the form of fat. Unfortunately, spontaneous regeneration and healing process for large bone defects is complicated due to their high complexity. Research carried out by Ng et al. have identified various cell sources ranging from stem cells to adult bone cells, and developed various scaffolds, ranging from biological tissues to synthetic hollow tubes to enhance in vitro and in vivo bone regeneration [1,2,3,4,5]. From the beginning of human civilization, ceramic has been as a bone substitute [6]. Calcium phosphate-based ceramics such as hydroxyapatite and calcium phosphate were the first to be used in the clinics as they make up the component of native bones. Other forms of ceramics that are biocompatible such as silicate, wollastonite, calcium sulfate, and calcium carbonate also found their way into the clinical setting as they offer similar osteoconductive and osteoinductive properties [7]. In general, these bioceramics are biodegradable although at different rates depending on their composition and structure [8,9,10,11]. From this we can summarise that bioceramics are able to degrade at different rates depending on their structure and composition.

However, a marked disadvantage of these materials is its brittleness [12]. This has driven most research toward the incorporation of polymers such as collagen, elastin, polycaprolactone to enhance its modulus strength [13]. Such an approach may be suited for non-load bearing bones as the process of bone regeneration and remodeling requires time before it can achieve the mechanical properties of native bone [14].

Metals such as stainless steel and titanium were seen as an obvious choice as bone implants due to their strength and bioinertness [15]. Their bioinert properties although advantageous if assessed from the perspective of an immune reactive scenario, however, make them non-responsive to its dynamic microenvironment. This has led to its poor integration with the host tissue, ultimately leading to implant loosening [16,17,18,19]. A combination of metal and ceramic seemed to be a perfect hybrid to cater to the mechanical loading and osteoinductive needs. One good example of this is titanium and wollastonite. Titanium wollastonite is a relatively novel material having the combination of titanium alloy (TI6Al4V) with wollastonite (CaSiO_3_). This material has good bioactive properties, and its mechanical strength has been shown to be similar to bone. Moreover, its response to stem cell viability show that it does not give off any negative responses when interacting with the material [20]. As this material is still new, more tests are required to be performed in order to properly assess its capabilities.

This paper focusses on the properties, fabrication techniques and in vitro, in vivo and economical evaluations relevant to the development of titanium ceramic as implants for bone tissue engineering. Table 1 and Table 2 highlight the pros and cons of the most common metals and bioceramics used as bone implants. Besides wollastonite which has only been evaluated in pre-clinical models, all materials listed are already in use clinically.

## 2. Methods

**Figure 1:**
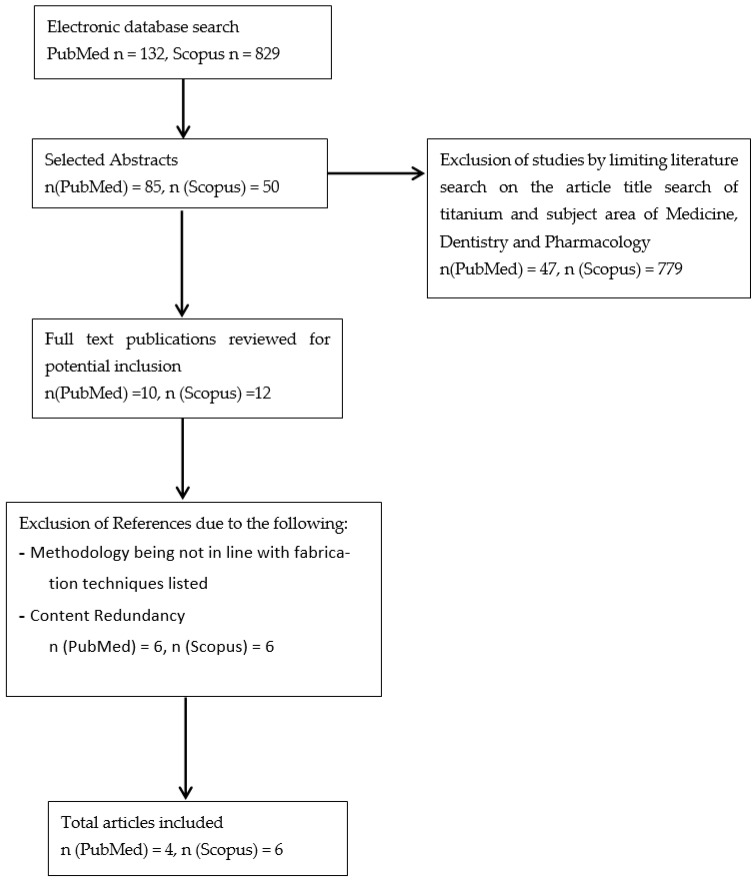
Flowchart of the Selection Criteria Process.

This review, which includes studies published from 1992 to 2019, presents an overview on recent progresses in the development of titanium–ceramic composite materials. Eligible studies were identified through an electronic search using Scopus and PubMed. From the results of refining the search from Scopus alone, only 6 papers were in line with the narrative of the review. From the refined results from PubMed only 4 papers were selected with the similar reason during the search refinement from Scopus. The purpose of this review is to provide a general evaluation of the current prospects of metal ceramic composites, primarily titanium, and exploring potential fabrication techniques and future clinical applications. Figure 1 and Table 3 give a further description of the search strategy as well as the eligibility criteria and its sources.

## 3. Structural Property of Metals and Bioceramics

### 3.1. Surface Characteristics

Metallic structural property would differ greatly from the ceramic group. The SEM images shown in Figure 1 presents a smoother surface on most of the metallic groups. The SEM images display smooth surfaces, and this presents uniformity within the material. Here a critical disadvantage is present, of which cells are unable to adhere to the smooth surface of the metals unless the metal has been modified to increase its roughness. Another problem is that no micropores are present in the metals hence in a way where it is porous. This means for the metal to be viable, modification of the surfaces is required to allow cells to vascularize from the porous structure of the material [31,56]. This is stressed further showing that surface roughness at lateral length scales slightly larger than the size of individual bacteria, such as those found in satin surface finishes would substantially enhance bacterial colonization as compared to polished or plasma-sprayed finishes, both of which are relatively smooth at microscopic length scales. This means that in order for the prevention of bacterial adhesion and for the development of osteogenic cells within the material, spreading and differentiation would have to be promoted by microscopically smooth and macroscopically rough surfaces which would be able to alter at length scales on the order of tens of microns corresponding roughly to the size of an individual tissue cell [57].

Angiogenesis which is the development of new blood vessels within the body also plays a key role in cellular integration giving the area of the implant an effective network [58,59]. This mechanism plays a key role in integrating the implant with the cells to ensure adequate supply of nutrients via the network of. Several researchers have demonstrated that the need for angiogenesis is crucial to the development of cellular integration [32,33]. In short, a porous structure allows angiogenesis to occur within the implant and further promotes osteointegration.

A porous structure is ideal in promoting cellular growth in 3-dimensional engineered tissue [8,9,34]. However, having a higher porosity or bigger pore size would also mean that the material has a weaker tensile strength and reduces the stability of the biomaterial [60,61]. This has also not only been shown in polymer-based biomaterials but also metals such as tantalum [35].

Porosity and pore size both in the macroscopic and the microscopic level, are crucial morphological properties for implant materials in order to facilitate to bone regeneration. Large pores averaging between 100 to 600 μm show substantial bone ingrowth, while smaller pores result in ingrowth of unmineralized osteoid tissue averaging between 75 to 100 μm and fibrous tissue averaging between 10 to 75 μm [62]. The minimum pore size required to regenerate mineralized bone is generally considered to be 100 μm [63].

**Figure 2:**
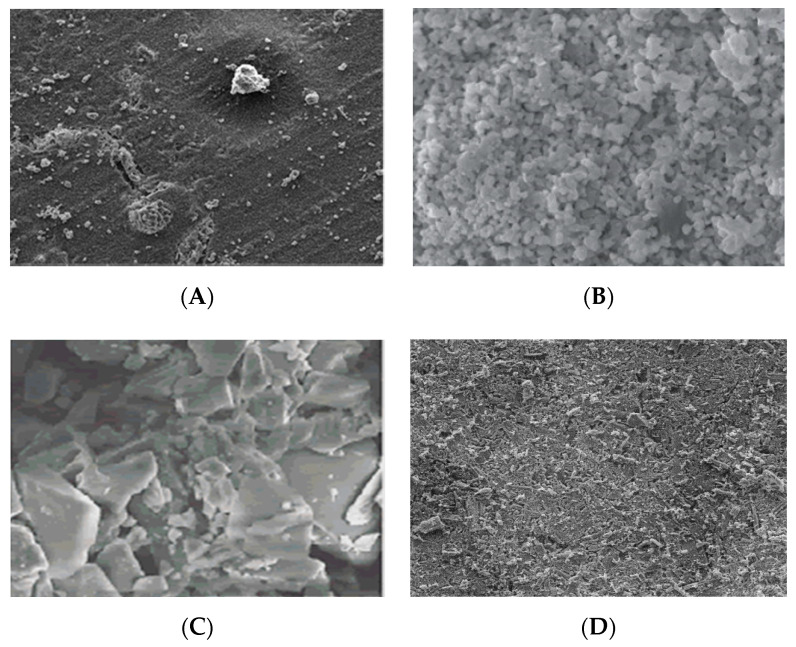
SEM images of bioceramics currently used for bone implants vs wollastonite (**A**) Hydroxyapatite, (**B**) Tricalcium Phosphate [69], (**C**) 45S5 Bioglass [70], and (**D**) Wollastonite.

Bioceramic is considered bioactive due to its surface roughness and apatite formation. In a general context, the rougher the surface, the more ideal the bioceramic would be able to support cellular attachment. Using the example of a composite of microhydroxyapatite and nanosilica, the ideal roughness of both material was recorded between the range of 525 nm to 725 nm [64]. This is further supported by using three different surface roughness of hydroxyapatite to identify cellular attachment and proliferation with the best roughness value for that study being the hydroxyapatite that was applied with a 180 grit sandpaper finishing as compared to a finer sandpaper using 1200 [65]. Figure 2 reveals the surface property of common bioceramics used in bone implants and wollastonite. These materials are also known as bioactive ceramics and what provides them their bioactive properties are their inherent ability to form a bond with bone tissue hence inducing osteogenesis [66]. The important component to note is that these ceramics must have at least calcium as their component as it is critical for the development of bone [67,68]. Another component that is beneficial includes phosphorus.

### 3.2. Mechanical Properties of Metals and Ceramics

An important approach in the field of biomaterials is to assess the quality and strength of the materials that are to be taken into consideration whenever fabricating a desired implant. Several studies that have been carried out have determined that the elastic and tensile strength is required to be taken into consideration whenever the implant has been fabricated to ensure that it can withstand the amount of load that is exerted by the area of implantation. This strength measured is recognized as known as Young’s Modulus and most biomaterials based on metals and ceramics have a higher Young’s moduli than cortical and trabecular bones [71,72,73,74]. Table 4 illustrates the Young’s Modulus for the notable metals used for bone implants. 

As for the bone’s young’s modulus, the idea of an implant development requires that the implant should be as close to the bone’s young’s modulus as possible if not lower. This is to avoid the side effect of stress shielding which will lower the bone’s density as the bone would no longer be dependent on the load placed by the body [77]. The table shown below highlights the moduli for both the cortical and trabecular bone. In summary, the Young’s modulus for the metals greatly exceeds that of the Young’s moduli of bone. One way to counter this and reduce the modulus of the metal is to reshape is as a thin rod or to reduce the metal’s size [78]. This, in turn, creates a limited amount of stress to that which the bone would still require itself to support. Even ceramics have to some degree a higher Young’s modulus than that of the human bone, which is shown in Table 5 for some of the commonly used ceramics in bone tissue replacement. The notable ceramics shown in Table 6 all have higher moduli than the cortical and trabecular bone, but they are biodegradable hence they will eventually match the strength of the bones given enough time.

## 4. Advantage of Metal-Ceramic Composites as Bone Implants

### 4.1. Physical Properties of Metal-Ceramic Composites as Biomaterials

Earlier the discussion concerning the importance of metals and ceramics as metals contain the strength to support our body and the efficiency of ceramics has their ability to generate bioactivity for the cells within their vicinity was implemented. This verdict was valid back then but today, the criteria for a good biomaterial implant include both the physical properties of the bone-implant material and its ability to promote the growth of body tissue. In a research conducted by Wang 2018, it is important to note that although titanium alloys and cobalt chromium alloys have been used have been successfully used in the use of orthopedic hip implants, there are still concerns over their clinic performance against corrosion, specially wear-assisted corrosion. This is why there is a growing need to involve metal implants with ceramics as standard metal based implant seem to be less popular in promoting bone growth and regeneration [93]. Metal-based implants tend to have a higher Young’s modulus than bone, which leads to stress shielding as was discussed in Section 3.2. Metal implants are also bioinert which means that cells do not react to them and merely encapsulate them, and this does not promote the growth of natural tissue. 

### 4.2. Efficiency of Titanium–Hydroxyapatite as a Metal-Ceramic Hybrid

Titanium–hydroxyapatite has been extensively used as a standard for most bone biomaterial implants. It not only possesses the strength of titanium but also the bioactivity that the hydroxyapatite can exhibit which in turn provides a stable implant. Hydroxyapatite also has a similar structure to the bone and helps to facilitate the growth of natural tissues. By combining a metal with a ceramic, a new biomaterial can be developed with excellent mechanical and biological properties. Multiple studies have confirmed the effectiveness of hydroxyapatite using titanium alloy as a base coating in order to boost bone growth and osteointegration. Thus we can state the significance of utilizing ceramics as a supplement of titanium alloy [94,95,96].

Conventional use of this hybrid is applied as a coating in which the ceramic acts as the coating to the metal base implant. This technique is achieved with the use of plasma spraying which is a complex process that involves rapid melting and high-velocity impact deposition of powder particles. One research carried out showed the use of coated hydroxyapatite through the plasma spraying method and shows that by choosing the specific initial powder composition and successive thermal treatment one can obtain HA/βTCP plasma sprayed coatings with tunable solubility for the selected biomedical applications [97]. The hydroxyapatite to be plasma sprayed onto an implant of pure metal converting itself from a powder form to a semi-liquid form creating a layer of the ceramic surrounding the metal base and this will allow the cells to benefit completely from the bioactivity of the ceramic while having the titanium to anchor the implant within the body. Another paper has also supported this method with one downside being that the material required further heat treatment in order to enhance the mechanical properties of the scaffold, therefore boosting its durability and strength [98].

In vitro tests have also shown that titanium–hydroxyapatite greatly supports osseointegration and osteogenic lineage regeneration as well [96,99,100,101] as in vivo tests, whereby the animal specimens achieved complete bone regeneration within several weeks [102,103,104].

### 4.3. The Prospect of Wollastonite in Metal-Ceramic Hybrid

Wollastonite, which is also known as CaSiO_3_, exists in two primary mineral phases, including β- wollastonite (wollastonite) and α-wollastonite (pseudowollastonite). The β-wollastonite mineral is obtained as a natural silicate mineral, whereas α-wollastonite is rarely found in nature. The β-wollastonite mineral phase is produced at lower temperatures than is α-wollastonite, which is apparent in the CaO–SiO_2_ binary phase diagram, in which the phase transition temperature of β- wollastonite to α-wollastonite is greater than 1125 °C [105,106].

Wollastonite is a versatile industrial mineral that is used in ceramics, cements, paints, paper, and plastics [107]. The versatility of wollastonite has generated much interest due to its promise as a potential implantable material because it can form bonds with bone tissue through the development of a biological apatite layer on the surface [10,108]. Materials with this characteristic are known as bioactive materials and are widely used in medical and dental applications. Many studies have been performed to produce bioactive materials for various applications, such as implantable materials [10,11,108,109,110].

Its application as a biomaterial is limited as not much research is done since many still prefer hydroxyapatite because of its closeness to the bone. Here we would like to stress that although not much research has been carried out on the prospect of wollastonite in terms of exploiting its bioactivity, it is important to note that wollastonite can act as a substitute to hydroxyapatite due to the shared composition of calcium contained within the material. It is also important to note the presence of silicon which has alone promoted the growth of bone which inevitably lead to osteointegration [111,112]. Economically speaking, its cost is also significantly lower that hydroxyapatite which will be further explained in part 6.

## 5. Fabrication of Metal-Ceramic Implants

### 5.1. Plasma Spraying as a Conventional Method

**Figure 3:**
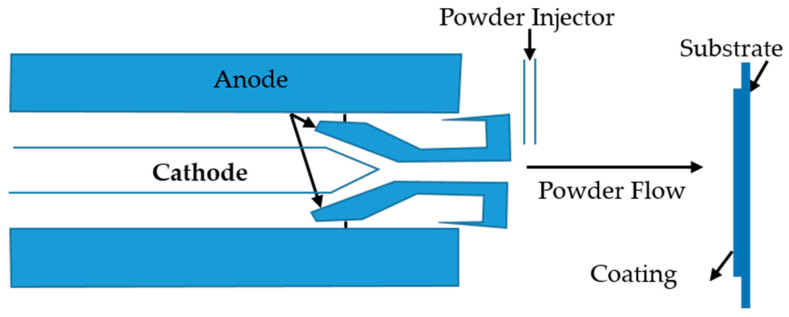
Simplified Overview of Plasma Spraying Process [116].

Plasma spraying is a well-established method for forming protective coatings and free-standing shapes from a wide range of alloys and ceramics. A complex process which involves rapid melting and high-velocity impact deposition of powder particles [113]. For a metal implant fabrication, this can sometimes involve a ceramic to be plasma sprayed onto an implant of pure metal. This will allow the cells to benefit completely from the bioactivity of the ceramic while having the titanium to anchor the implant within the body [114]. Figure 3 shown below represents an image of the plasma spraying process that takes place [115]. Initially argon gas flows between the electrode and nozzle. The ionization of the gas stream is carried using a high voltage alternating electric arc. Increasing the arc current, helps it to thicken and increase the degree of ionization. This has the effect of increasing the power and, the velocity of the gas stream.

Once the appropriate gas stream has been established for the material being sprayed, the feedstock which typically comes as material in various powders is injected into the gas stream [115]. This will create the spraying effect which will coat itself onto the material of choice.

### 5.2. Powder Injection Moulding

**Figure 4:**
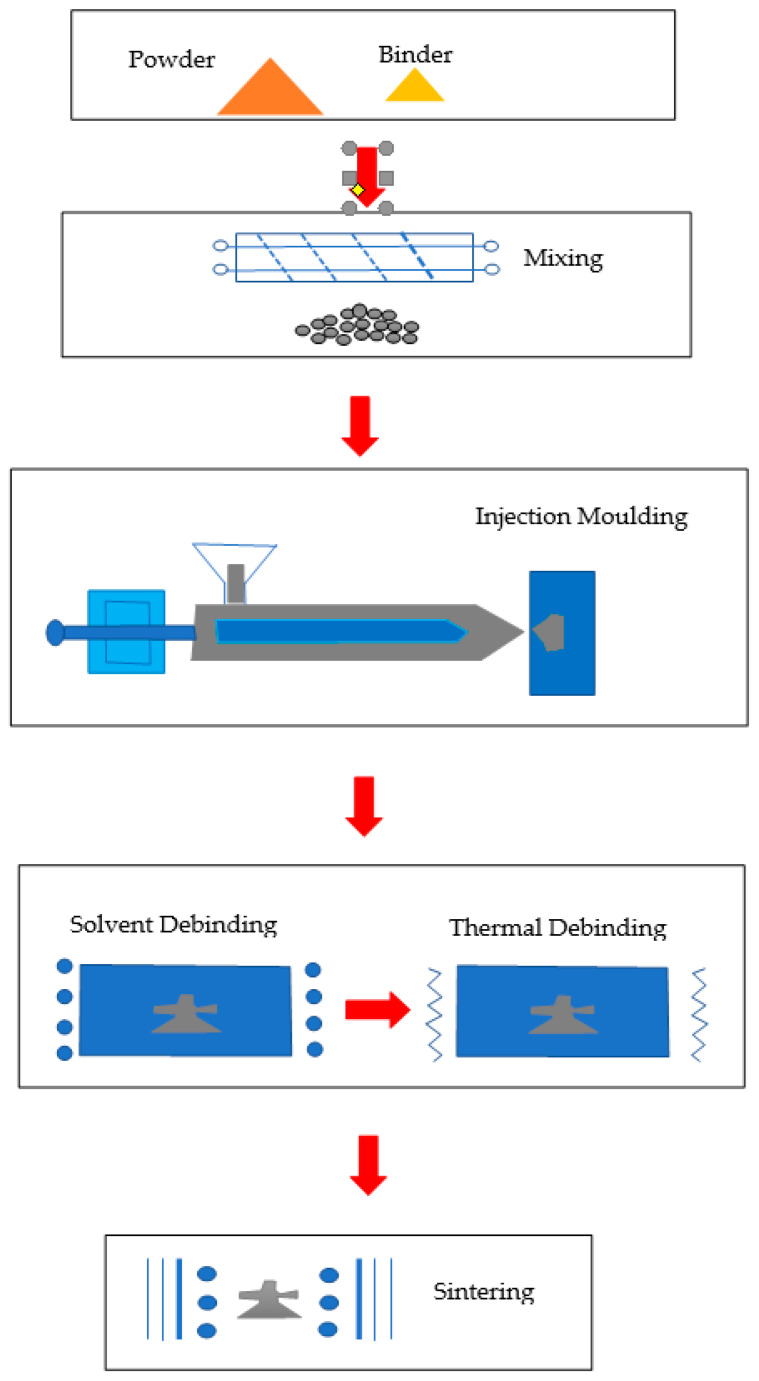
Overview of Powder Injection Moulding Process [121].

Apart from the conventional methods discussed earlier, other methods have been employed to fabricate these metallic biomaterial structures. One such study has shown that titanium–ceramic fabrication is possible using binders before sintering. This uses a PIM (Powder Injection Moulding) machine and it uses two different types of powders; one metal and one ceramic which then they are mixed with the palm stearin and polyethylene binder system before they are compressed in a powder injection molding machine whereby under high pressure they are extruded out from a nozzle and enter a mold casing with a specific shape [117,118]. Other studies have also been conducted using the PIM technique to manufacture titanium-based scaffolds for implant use [119,120]. Figure 4 shows the process of powder injection molding whenever powder is loaded into the mold cast. One research done by our collaborators have found that the behaviour of their Titanium alloy-Wollastonite feedstock show pseudoplastic behaviour; where viscosity decreases with increasing shear rate and temperature, making it ideal for this process [20].

### 5.3. Powder Compaction

**Figure 5:**
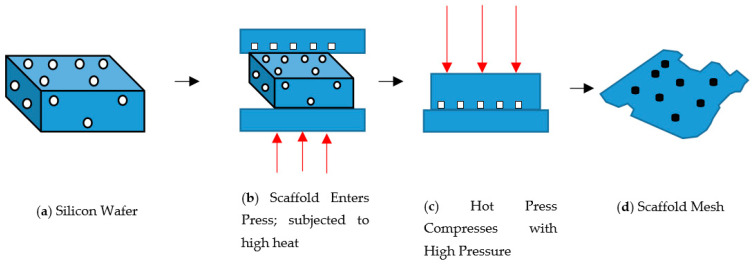
Overview of Compression method for Metal-Ceramic Scaffold Fabrication [128].

Powder compaction is the act of using a piston-like instrument to press down a substance, forming a compressed material. This compacts the powder in a die through the application of high pressures. Typically, the tools are held in the vertical orientation with the punch tool forming the bottom of the cavity. The powder is then compacted into a shape and then ejected from the die cavity [122]. This method has only begun to recently surface as not many people were aware of this method of fabrication and that implant manufacturing [123,124,125,126,127]. The premise is that with the powder compaction method, this method would allow us to form a composite containing both metal and ceramic and also, once the ceramic portion of the material degrades from the implant, the metal implant would become porous and this will enhance further vascularisation and migration of cells towards the inner recesses of the implant. Figure 5 shows a schematization of the fabrication process for 3D PCL pillared implants using a hot press (on the left). Image (a) represents Silicon master production, (b) represents micro-moulding melting step. Image (c) micro-moulding pressing step and (d) is the final structure obtained after solidification and detachment. We are currently doing our own study on titanium alloy-wollastonite using this process and found that a compaction force of 10 kg/m^3^ at 120 °C is suitable when using a cylindrical stainless steel mould measuring 12 mm in height and 15 mm in diameter.

Additive manufacturing is a novel materials processing approach to create parts or prototypes layer-by-layer directly from a computer aided design (CAD) file. It is important to note that there is some significance in the development of biomaterials using this technique as it has been done with some degree of success [129,130,131,132]. This allows further flexibility in the process of manufacturing implants for biomedical purposes.

## 6. Minimal Evaluations Required for New Materials before Clinical Applications

### 6.1. In Vitro Evaluation

Once various implants have been fabricated, they will usually enter in vitro studies before moving on to in vivo based studies. Some of these studies have been potent within the development of implants for cellular regeneration. An important aspect to look at is the biocompatibility of the implant concerning trigger a cellular response. One study has demonstrated that using a hybrid of Magnesium and hydroxyapatite has significantly improved in osteogenic cell adhesion and viability, which emphasizes its candidature as an orthopedic accessory [133]. Biocompatibility has been the gold standard for any type of implant be it metal, ceramic or even polymer. Another study using calcium titanate coating on titanium screws has also demonstrated the biocompatibility of the material such that it has excellent synergistic properties with osteoblastic cells [134]. Here we can summarise that knowing and identifying whether metal-ceramics are biocompatible represents a crucial first step for the material to be identified as a biomaterial. Another important aspect to consider within the in vitro analysis is the ability of the osteoblasts to proliferate and integrate within the implant. Table 7 shows a list of comparisons of different titanium–ceramic implants and how their in vitro work has contributed to the development of metal-ceramic implants.

A study that was carried out has successfully demonstrated the efficacy of osteoblasts in creating an ossification action towards the implant; encapsulating them [135,136]. Central to in vitro studies are also the importance of cellular immune responses between implant and cell. Many studies have been carried out and have yielded consistent results regarding immune responses from cells towards metal-ceramic implants. We have discussed in length that most metals have an inert characteristic, therefore, they do not trigger immune responses but merely let cells encapsulate them and as for ceramics, they tend to have synergistic properties as they are bioactive. In the case of metal-ceramic implants, macrophages tend to act as osteoclasts as one study has proved this [135]. This, in turn, creates an effective immunomodulatory environment between the implant and its area of implantation.

### 6.2. In Vivo Evaluation

Whenever the functionality and biocompatibility of these biomaterials are considered, there are assumptions that these materials can promote a significant amount of cellular biocompatibility within a host body. However, this may be inaccurate as several studies have shown losses in biocompatibility due to the formation of acidic degradation products that alters the activity of mesenchymal stem cells which then leads to triggering inflammatory reactions [137,138,139,140]. Moreover, concerns have been raised which implies the presence of residual organic solvents used in the manufacture of the implant [141].

Although polymers are more likely to cause problems associated with biomaterials, metals have their challenges such as ionic degradation as well as its metal particulates which accumulate in the bloodstream which have been examined in magnesium-based implants [142]. Titanium, however, does not do this. However, it also cannot ease bone attachment due to its inert nature [143]. Stress shielding is also a common occurrence in the usage of these metal types. Stress shielding refers to the act of the density of bone decreasing due to the larger tensile strength on the biomaterial implanted within it. Our bones are required to have some form of load to promote a stimulus to keep its density intact. If the material’s modulus is greater than the bone’s, this would result in an osteopenia [77]. This can lead to more complications which is why the bone must be the one that maintains most of the support.

## 7. Commercialized Metal-Ceramic Implants

The commercialized products regarding titanium–ceramics have been well established by several companies. Table 8 shown below summarises various orthopedic companies and their products within the biomaterials sector. Most of the companies employ the use of titanium for base implants in producing hip replacements, except for bioimplants made by Stryker and Smith and Nephew. In short, these companies have commercialized the product of a biomaterial and utilized their biocompatibility in various orthopedic and spinal applications.

In a more retrospective analysis, several studies have pinpointed the rising costs of total knee replacements. Implants that were used in revision total knee arthroplasty contributed a significant proportion of the total hospital cost of admission for the care that had been provided. The analysis was that by using a standardized model or a fixed model, direct to hospital implant pricing could potentially save approximately $7000 per revision total knee arthroplasty, and the fixed pricing model could provide a further $1000 reduction per revision total knee arthroplasty potentially saving $8000 per case on implants alone based on the average cost for revision of all components which was $13,640 and ranged from $3000 to $28,000. On average, this represented 32.7% of the total hospital cost using the statistics of 52 patients [164]. This is further supported by the shift in healthcare costs on orthopaedic surgical procedures as a fixed payment system may close the gap between the cost of the implant and the rising costs of healthcare [165,166]. 

### Cost of Materials to Manufacture Implants

Within the development of implants, materials cost can play a key role in determining the implant’s feasibility to the public. With titanium costing approximately the same price with minor differences in prices between regions, the swing factor comes to the manufacturing of ceramics and polymers to complement the implant. For the justification of the cheapest implant, Table 9 shows the cost of hydroxyapatite based on a list of manufacturers from China and a price comparison between 2 key ceramics that were discussed in this paper; hydroxyapatite and wollastonite which is displayed in Table 10. From the information in Table 9 and Table 10, it clearly shows that the cost of hydroxyapatite is more expensive than wollastonite by a thousand-fold. Although hydroxyapatite has been effectively used within the implant industry alongside titanium, the incorporation of wollastonite can greatly improve the capital costs of implant manufacture.

Now a more interesting question would be should implants be permanent or removed? This has raised several issues in terms of the cost of removing the implant vs the cost of keeping it. A study carried out showed that the removal of implants itself carried a high-cost amount [173]. Table 11 adapted from the paper demonstrates this. This can also affect the hospital costs for implant procedures are high. Between the year of 2000–2004, knee arthroplasty was one of the top ten commonly performed procedures, with the most rapidly increasing hospital inpatient costs for all payers. Hip replacement was a top-ten commonly performed procedure with the most rapidly increasing inpatient costs for private insurance [173]. In 2004 there were 488,000 hospital stays in U.S. hospitals for knee arthroplasty procedures, with mean length-of-stay of 3.9 days and mean cost of $13,200 per admission. In the same year, there were 368,000 total and partial hip replacements, with mean length-of-stay of 5.0 days and mean cost of $14,500. Aggregate costs were $6.3 billion for knees and $5.3 billion for hips [173].

## 8. Recommendations

The development of implants is crucial to the progress of implant improvements made from materials to be as close as the properties of the human body as possible. Materials developed should also be able to address the situation of the targeted area. For example, a material designed to treat bone should contain at least calcium or phosphorus to allow cells to recognize and adapt to the implant. In hindsight, most of the material that we use to develop implants and implants are not fully utilized to their fullest potential. This is also mainly because there is still little research carried out to exploit the beneficial properties of everyday materials and their synergy with our bodies. This review article is written to encourage readers to be able to understand the advantages surrounding composite hybrid implants and how they can be greater than implants fabricated through conventional means by layering metal or ceramic on top of another metal.

## 9. Summary

In conclusion, to select a good metal-ceramic biomaterial, the biomaterial needs to be able to follow a certain number of standards. Firstly, the biomaterial is required to be either bioinert, biotolerant, or bioactive to achieve a good biocompatibility and needs to have a good selection of metals and ceramics, its fabrication methods can be achieved either via conventional means or even through methods such as compaction. It should also have good osseointegration and able not to provoke a negative immune response when implanted. The biomaterial should also have its commercial value unprecedented, although it does have its obstacles when undergoing this type of challenge within the medical field in addition to the rising medical costs for such technology.

## 10. Future Directions

As observed from the studies mentioned above, the effect of various metal-ceramic materials has been proven to be osteoconductive in animal models and humans. However, more studies are still required to identify the mechanism of the cellular interaction between the titanium–ceramic as well as its biocompatibility with the extracellular matrix within the human body. Besides that, the material’s properties are needed to be ascertained and standardized by more scientific studies. Furthermore, the effects of the bone cells on the titanium–ceramic in vitro as well as the in vivo conditions still yet remained to be explored. Thus, further studies on the effect of the human bone cells on titanium–ceramic are required to be undertaken to greatly enhance its novelty, as well as to accelerate its economic potential as well as its economic value in the field of clinical science.

## Figures and Tables

**Table 1:** Characteristics and Limitations of Current Metallic Biomaterials.

Metals	Advantages	Disadvantages
Titanium Alloy (Ti 6Al-4V)	Lightweight, corrosion-resistant, very biocompatible. Forms layer of titanium oxide, which is a stable and reactive interface that becomes coated with plasma proteins [21].	Expensive Can cause an allergic reaction when the body is exposed to very cold weather [14] Cannot be used as the ‘ball’ role in the ball and socket joint, as friction can easily form. This can be avoided by reducing the roughness of the material so that it provides a fail-safe running function in case of material debonding [22]. Susceptible to implant loosening [23]
Stainless Steel 316	Low carbon diminishes corrosion Decreases adverse tissue responses and metal allergies [24]	High corrosion rates make it a poor candidate for the manufacture of modern joint replacement implants [24] Low osseointegration due to its bioinert properties similar to Titanium alloy [21]
Cobalt-Chromium	It can be used in various porous forms to allow for biologic fixation by ingrowth. High-temperature resistance [25]. Well suited to produce implants that are designed to replace bone and to be load-bearing for an extended period, if not permanently [26]	Least ductile when compared to either iron- or titanium-based alloys, making manufacturing them more difficult [22]. Very high moduli of elasticity, which leads to stress shielding [27].
Nickel-Titanium(Nitinol)	Has superelastic and shape memory properties which allow lower fatigue than most other metallic alloys [28] Forms titanium oxide which further enhances biocompatibility [29]	Release rate of nickel into the body has to be limited as it can cause toxicity to the body if not controlled properly [30]
Niobium-Zirconium	Very high corrosion resistance and corrosion fatigue [31] Air oxidation forms oxidized zirconium which is shown to have good biocompatible properties [32]	Low critical stress slip for Niobium would require heat treatment in order to increase mechanical stability for material [33]
AZ91D	Alloy is made of Magnesium and has high biocompatibility [34] High fatigue resistance allowing material to have lower tendency to wear and tear [35]	Magnesium has poor corrosion resistance hence requiring anti-corrosion coating [36]

**Table 2:** Characteristics and Limitations of Current Ceramic Biomaterials.

Ceramics	Advantages	Disadvantages
Hydroxyapatite	Develops tight bonding with bone [26]. Very good integration as cells would be able to benefit from its bioactivity [26].	Placing and retaining the particulate within the area of defect [37] A large amount of time is needed to fully restore bone [37]. Material by itself is very brittle [22]
Tricalcium Phosphate	Contains calcium and phosphate which assist in bone integration and bone formation [38] Best graft for bone compared to other synthetic grafts [39]	Very brittle, poor fatigue strength and fatigue resistance [40]
45S5 Bioglass	It can bond both soft and hard tissues [41]. Its bioactivity allows integration with cells [41] Can be molded easier than other composite ceramics [42]	Brittle and fragile [43] Generally restricted to clinical non-load bearing situation [43] Causes ankylosis and decreased fracture resistance [44]
Wollastonite	High bioactivity [45] Faster apatite formation than most other ceramics [46]	High degradation rate than other ceramics [47] Must be incorporated with another material (ceramic or metal) to be viable [48]
Calcium Sulphate	Well known as a primary material for plaster of paris and it has served well as bone defect filler [49] Shows good biocompatibility and osteointegration [49,50]	Structural support is severely lacking [51] Potential for antibiotic losses through wound discharge [52]
Calcium Carbonate	Low cost and biocompatible as well as being highly osteoconductive [53] Very high resorption rate allowing fast replacement of bone tissue [54]	Has high aqueous instability which leads to bust release of its payload preventing proper controlled rate of release [55]

**Table 3:** A brief summary on some of the articles included.

No	Articles (Database)	Type of Metal	Type of Ceramic	Methodology	Results	Conclusion
1	Spoerke 2005 (Scopus)	Commercially pure Titanium powders (particle size 130 µm)	Organoapatite (OA) which consists of hydroxyapatite mineral, which has been precipitated in the presence of macromolecules incorporating these macromolecules into the mineral phase.	**Treatment Groups** Bare Titanium Foam Titanium combined with Organoapatite **Parameters** Characterisation was carried out using FTIR and SEM Alkaline phosphatase was quantified by liberating the DNA from cells and were combined with Hoechst 33258 in solution and quantified by fluorescence on an ISS PC1 fluorescent spectrophotometer Osteocalcin was qualitatively identified by cellular trypsinisation from foam substrates and plated onto tissue culture polystyrene well-plates. After 8 h cells were fixed at 4 °C for 1 min with a solution of 70% ethanol, 25% distilled water, and 5% acetic acid. Fixed cells were rinsed in phosphate buffered saline (PBS) and incubated for 30 min in 1% bovine serum albumin (BSA). Double antibody immunohistochemistry was then performed using goat anti-mouse osteocalcin as the primary antibody and a fluorescently tagged donkey anti-goat secondary antibody In vivo assessment was carried out by basing approximations from the experimental data collected in the experiment, a commercial FE software package (ABAQUS Standard 6.3-1) was used to create two- dimensional meshes, representative of 25% porous foam microstructures with 25 round pores, whose size and position were randomly perturbed from an average value and a regular 5 × 5 array, respectively. Pores were selectively filled with inclusions with the mechanical properties of bone to simulate the effects of bone in- growth within the pores of the foam structure. Foam constructs were sandwiched between model layers of bone, representing bony tissue adjacent to an implant. Four mesh structures were utilized in the FE simulations: foam with empty pores, foam where outer pores only were filled with bone simulating partial bone ingrowth, foam where all pores were filled with bone, and solid titanium	FTIR scans confirmed this to be an apatitic structure with characteristic bands at 567, 605, 964, 1037, and 1100 cm^−1^. Poly(L-lysine) present and associated with the apatitic phase was also indicated by an amide I band (C=O stretch) at 1650 cm^−1^ Alkaline phosphatase shows that by 14 days of cellular colonization, the preosteoblastic cells had begun to upregulate their alkaline phosphatase expression, indicating osteoblastic differentiation. This effect increased dramatically by 28 days. Osteocalcin was also expressed in cells from OA-coated foams after 28 days of culture. There was no apparent influence of the OA coating on the rate or intensity of osteocalcin expression The FE modeling showed that porous models were completely elastic up to 0.11% uniaxial external strain (0.14% for the fully bone-filled model), whereas the solid titanium model was completely elastic through 0.25% strain. The elastic modulus calculated from the FE analysis of the foam constructs was 57.4 GPa	In vitro experiments in a rotating bioreactor demonstrated early colonization of organoapatite-coated titanium foams by preosteoblasts
2	Chu 2004 (Scopus)	Titanium alloy powder (Ti 99.3%, Fe 0.039%, O 0.35%, N 0.035%, C 0.025%, CL 0.034%, H 0.024%, and Si 0.0018%) with mean particle size of 45.2 µm	Hydroxyapatite powder prepared by the reaction between Ca(NO_3_)_2_ and (NH_4_)_2_HPO_4_.	**Treatment Groups** Titanium Hydroxyapatite (40% volume Hydroxyapattie and 60% volume Titanium) Titanium Hydroxyapatite (20% volume Hydroxyapattie and 80% volume Titanium) Hydroxyapatite **Parameters** Characterisation was carried out using XRD, SEM, Vickers Hardness test and 3 point bending tests In vivo study by drilling into several New Zealand white rabbit’s skulls and implanting the specimen	XRD data shows that hydroxyapatite and titanium phases are the predominant phases of the composite like HA 20 vol.% Ti composite. In addition, the sintering process at a high temperature resulted in the formation of two secondphase, such as a α- Ca_3_(PO_4_)_2_ (TCP) and Ca_4_O(PO_4_)_2_. SEM data shows obvious Ti metal particles homogeneously distributed in HA ceramic matrix. Vickers and the 3 point bending tests reveal HA 40 vol.% Ti composite with similar elastic modulus (79.3 GPa) and Vicker’s hardness (2.94 GPa) has a higher bending strength (92.1 MPa) In vivo results show that HA 40 vol.% Ti composite has excellent biocompatibility and could integrate with bone	The most effective group of was the Titanium Hydroxyapatite (40% volume Hydroxyapattie and 60% volume Titanium) as it was proven to be effective in the in vivo study as no fibrous tissues came into being at the interface between the composite implant and newborn bones
3	Peñarrieta-Juanito 2018 (PubMed)	Commercially pure titanium Grade V	Hydroxyapatite and Beta Tricalcium Phosphate	**Treatment Groups** Titanium Hydroxyapatite Titanium Beta-Tricalcium Phosphate Commercially pure titanium **Parameters** Characterisation through shear bond strength tests, surface roughness analysis, water contact angle measurement and SEM. Cell viability was done through Cell-Titer Blue buffer Bone markers were identified using an Alkaline Phosphatase Kit.	Shear bond strength of titanium showed the highest mean values of shear bond strength at about 548 MPa considering there is no transition zone within bioactive ceramic. Average roughness (Ra) values were quite similar among the groups although titanium revealed the highest water contact angle (WCA) values which were 111.9° Cell viability results show the control group (well plates) showed the highest values of cell viability, due to the high roughness of the polystyrene based material which is proper for cell culture. However, titanium, titanium hydroxyapatite, and Titanium beta tricalcium phosphate samples possessed similar roughness mean values for comparison of results concerning the differences in chemical composition of the test surfaces For the Alkaline phosphatase results, test groups containing bioactive materials had higher cellular activity when compared to that recorded for Titanium group	The titanium beta-tricalcium phosphate composite provided a higher viability and alkaline phosphatase activity with noticeable formation of mineral matrix when compared to commercially pure titanium containing or not containing hydroxyapatite
4	Ramli 2019 (Pubmed)	Titanium alloy(Ti6Al4V) powder	Wollastonite (CaSiO_3_) created from rice husk ash	**Treatment Groups** Titanium Wollastonite **Parameters** Rheological properties were evaluated based on the viscosity and shear rate sensitivity using the feedstock at different temperatures of 130 °C, 150 °C and 170 °C. The density of the sintered part was assessed using a conventional liquid displacement method according to Archimedes’ principle. They were analysed using sintered temperatures of 1100 °C, 1200 °C, and 1300 °C Charaterisation material using Young’s Modulus was obtained from 3 point bending tests as well as EDX. Cell viability was also assessed using PretoBlue reagent	Titanium wollastonite rheological property showed the feedstock has a pseudoplastic behaviour and it is desirable during the moulding process From the density data it is shown that when the sintering temperature increased, the density also increased. Furthermore, the sintered part becomes denser when the temperature increased to 1300 °C which was 4.12 g/cm^3^. The largest Young’s modulus value is obtained at 1100 °C at 18.10 GPa and as the temperature increased, the modulus also decreased although it is still acceptable to be used for bone implant applications; since it is within the Young’s modulus range for human bones (10–30 GPa). EDX data shows high oxygen content on the material sintered at 1100 °C. A higher oxygen content can increase the strength of sintered part. Cell viability results show that all the materials sintered at different temperatures are not cytotoxic and have good biocompatibility after 7 days.	Titanium wollastonite composite fabricated at a sintering temperature of 1100 °C is biocompatible and contains bioactive properties for bone implant applications

**Table 4:** Young’s Modulus of metals used as bone implants.

Metals	Young’s Modulus	Compressive Strength	Tensile Strength
Titanium 6Al-4V [75]	114 GPa	1119 MPa	940 MPa
Stainless Steel 316 [76]	26.67 GPa	110.33 MPa	74.67 MPa
Cobalt-Chromium [75]	283 GPa	1976 MPa	1403 MPa

**Table 5:** Young’s Modulus of both Cortical and Cancellous (Trabecular) bone.

Bone Type	Modulus of Elasticity (GPa)	Compressive Strengh	Tensile Strength
Cortical	10–30 GPa [79]	141.6 MPa [80]	39.74 MPa [80]
Trabecular	4.5–23.6 GPa [81]	2.270 MPa [82]	4.5 MPa [83]

**Table 6:** Young’s Modulus on several popular ceramics that are used as implants.

Ceramic	Modulus of Elasticity	Compressive Strength	Tensile Strength
Hydroxyapatite [84]	119.5 GPa	520 MPa [85]	190 MPa [85]
β-Tricalcium Phosphate [86]	23 GPa	159.4 MPa [87]	0.17 MPa [88]
Wollastonite [89]	23 GPa	250 MPa	7.4 MPa [90]
45S5 Bioglass [91]	78 GPa	0.4 MPa	0.011 MPa [92]

**Table 7:** Various titanium–ceramic implants and their in vitro analysis.

Titanium–Ceramic Type	Genetic Expression	Cellular Attachment and Proliferation
Titanium Pure21	Large amounts of osteopontin were expressed initially, slowed overtime	Strong cell attachment weak proliferation
Titanium Hydroxyapaptite21	Moderate amounts of osteopontin expressed initially; increased over time until the 21st day then reduced	Strong cell attachment and proliferation
Titanium Silicanite21	Moderate osteopontin expressed; no gain or reduction overtime	Strong cell attachment and proliferation

**Table 8:** Various spinal and orthopedic companies specializing in bone replacement.

Company Name	Targeted Customers	Product Examples	Material Composition
Stryker	Biomaterials, trauma and spinal implant sectors	AlloCraft CA [144], BIO Wedge [145], ReUnion RSA [146], ReUnion S [147], T2 Humeral [148]	Femoral Allograft (freeze dried), Ti6Al4V
Zimmer Biomet	Artificial knees, artificial hips, extremities, trauma products spine	OsseoTi^®^ Porous Metal Tech [149], Versa-Fx^®^ II Femoral Fixation [150], NexGen^®^ Legacy^®^ Constrained Condylar Knee (LCCK) [151], Avenir^®^ Hip System [152], TrellOss™-C [153]	Ti6Al4V, Hydroxyapatitecoating, Cobalt-Chromium
DePuy Synthes	Arthroplasty, fixation, trauma and orthopedic solutions	ACTIS^®^ Total Hip System [154], ATTUNE^®^ Revision Knee System [155], GLOBAL^®^ CAP Conservative Anatomic Prosthesis and GLOBAL^®^ CAP CTA™ Cuff Tear Arthropathy Conservative Prosthesis [156], SIGMA^®^ Total Knee System [157], PROTI 360°™ Ti Integrated Technology [158]	Ti6Al4V, Hydroxyapatite coating, Stainless Steel 316 L
Smith and Nephew	Hip and knee implants,	Redapt [159], Verilast [160], Polar3 [161], Biosure HA and PK [162], Cargel [163]	Ti6Al4V, Stainless Steel 316 L, Stainless Steel 304 L, Bone Allograft, Hydroxyapatite coating

**Table 9:** Cost of Hydroxyapatite in Various Companies.

Company Name	Cost per Kilogram
Xi’an Lyphar Biotech Co., Ltd.	US $30–350 [167]
Xi’an Sgonek Biological Technology Co., Ltd.	US $50–80 [168]
Shanghai Ximeng New Materials Technology Co., Ltd.	US $180–250 [169]

**Table 10:** Cost of Wollastonite in Various Companies.

Company Name	Cost per Metric Tonne
Richin International Trade (Dalian) Co., Ltd.	US $120–140 [170]
Shanghai CNPC Powder Material Co., Ltd.	US $70–200 [171]
Shenyang Huakuang Trading Co., Ltd.	US $113–198 [172]

**Table 11:** Measured Economic Indices for Orthopaedic Implant Removal.

Index	Category of Patients	Mean Cost	Total
Cost	All Patients (n = 47)	$ 708.37	$ 33,293.59
	Symptomatic (n = 6)	$ 613.15	$ 3678.90
Cash that cannot be saved	$ 29,614.69		
Time Lost (Days)	All Patients (n = 47)	15.4	723
	Symptomatic (n = 6)	16.3	98
Time that cannot be saved (days)	625		

Source: [173].

## Data Availability

No new data were created or analyzed in this study. Data sharing is not applicable to this article.

## References

[1] Ng M.H., Duski S., Tan K.K., Yusof M.R., Low K.C., Mohamed Rose I., Mohamed Z., Bin Saim A., Haji Idrus R.B. (2014). Repair of segmental load-bearing bone defect by autologous mesenchymal stem cells and plasma-derived fibrin impregnated ceramic block results in early recovery of limb function. Biomed. Res. Int..

[2] Haji Idrus R.B., Abas A., Ab Rahim F., Saim A. (2015). Clinical translation of cell therapy, tissue engineering and regenerative medicine product in Malaysia and its regulatory policy. Tissue Eng. Part A.

[3] Tan K.K., Tan G.H., Shamsul B.S., Chua K.H., Ng M.H., Ruszymah B.H., Aminuddin B.S., Loqman M.Y. (2005). Bone graft substitute using hydroxyapatite scaffold seeded with tissue engineered autologous osteoprogenitor cells in spinal fusion: Early result in a sheep model. Med. J. Malays..

[4] Sulaiman S.B., Keong T.K., Cheng C.H., Saim A., Bin H.J., Idrus R.B. (2013). Tricalcium phosphate/hydroxyapatite (TCP-HA) bone scaffold as potential candidate for the formation of tissue engineered bone. Indian J. Med. Res..

[5] Ng A.M.H., Tan K.K., Phang M.Y., Aziyati O., Tan G.H., Isa M.R., Aminuddin B.S., Naseem M., Fauziah O., Ruszymah B.H.I. (2008). Differential osteogenic activity of osteoprogenitor cells on HA and TCP/HA scaffold of tissue engineered bone. J. Biomed. Mater. Res. Part A.

[6] Yu X., Zhou W., Liu X., Xian F., Liu Z., Zheng Y., An Z. (2010). Peat records of human impacts on the atmosphere in Northwest China during the late Neolithic and Bronze Ages. Palaeogeogr. Palaeoclimatol. Palaeoecol..

[7] Dorozhkin S. (2011). Medical application of calcium orthophosphate bioceramics. Bio.

[8] Reza E., Aidin S., Reusmaazran Y., Rusnah M., Ng M.H. (2012). Fabrcating and evaluating the different ratio of TCP:HA as scaffold for bone tissue engineering. J. Tissue Eng. Regen. Med..

[9] Sadeghilar A., Ng A., Hwei M., Haflah N.H.M., Rose I.M., Firouzi S. (2014). Local tissue reaction and biodegradation of Hydroxyapatite/Tricalcium Phosphate composites. World J. Med. Sci..

[10] Shamsudin R., Abdul Azam F.A., Abdul Hamid M.A., Ismail H. (2017). Bioactivity and cell compatibility of β-wollastonite derived from rice husk ash and limestone. Materials.

[11] Azam F.A.A., Shamsudin R., Ng M.H., Zainuddin Z., Hamid M.A.A., Rashid R.A. (2018). Mechanical and bioactive properties of mullite reinforced pseudowollastonite biocomposite. Sains Malays..

[12] Le Huec J.C., Schaeverbeke T., Clement D., Faber J., Le Rebeller A. (1995). Influence of porosity on the mechanical resistance of hydroxyapatite ceramics under compressive stress. Biomaterials.

[13] Hannink G., Arts J.J.C. (2011). Bioresorbability, porosity and mechanical strength of bone substitutes: What is optimal for bone regeneration?. Injury.

[14] Marsell R., Einhorn T.A. (2012). The biology of fracture healing. Injury.

[15] Saini M. (2015). Implant biomaterials: A comprehensive review. World J. Clin. Cases.

[16] Aubret S., Merlini L., Fessy M., Besse J.L. (2018). Poor outcomes of fusion with Trabecular Metal implants after failed total ankle replacement: Early results in 11 patients. Orthop. Traumatol. Surg. Res..

[17] Takagi M. (2001). Bone-implant interface biology. Foreign body reaction and periprosthetic osteolysis in artificial hip joints. J. Clin. Exp. Hematop..

[18] Jiang Y., Jia T., Wooley P.H., Yang S.Y. (2013). Current research in the pathogenesis of aseptic implant loosening associated with particulate wear debris. Acta Orthop. Belg..

[19] Jiang Y., Han Y., Wang J., Lv F., Yi Z., Ke Q., Xu H. (2019). Space-oriented nanofibrous scaffold with silicon-doped amorphous calcium phosphate nanocoating for diabetic wound healing. ACS Appl. Bio Mater..

[20] Ramli M.I., Sulong A.B., Muhamad N., Muchtar A., Zakaria M.Y. (2019). Effect of sintering on the microstructure and mechanical properties of alloy titanium-wollastonite composite fabricated by powder injection moulding process. Ceram. Int..

[21] Brie I.C., Soritau O., Dirzu N., Berce C., Vulpoi A., Popa C., Todea M., Simon S., Perde-Schrepler M., Virag P. (2014). Comparative In Vitro study regarding the biocompatibility of titanium-base composites infiltrated with hydroxyapatite or silicatitanate. J. Biol. Eng..

[22] Marx R., Faramarzi R., Jungwirth F., Kleffner B.V., Mumme T., Weber M., Wirtz D.C. (2009). Silikatbeschichtung zementierter Titanschäfte für die Reduzierung aseptischer Lockerungsraten Silicate Coating of Cemented Titanium-Based Shafts in Hip Prosthetics Reduces High Aseptic Loosening. Zeitschrift Orthopädie Unfallchirurgie.

[23] Learmonth I.D., Young C., Rorabeck C. (2007). The operation of the century: Total hip replacement. Lancet.

[24] Yoo Y., Jang S., Oh K., Kim J., Kim Y. (2007). Influences of Passivating elements on the corrosion and biocompatibility of super stainless steels. J. Biomed. Mater. Res. Part B Appl. Biomater. Off. J.Soc. Biomater. Jpn. Soc. Biomater. Aust. Soc. Biomater. Korean Soc. Biomater..

[25] Hyslop D.J.S., Abdelkader A.M., Cox A., Fray D.J. (2010). Electrochemical synthesis of a biomedically important Co—Cr alloy. Acta Mater..

[26] Suchanek W., Yoshimura M. (1998). Processing and properties of hydroxyapatite-based biomaterials for use as hard tissue replacement implants. J. Mater. Res..

[27] Points P. (2017). Allergic reaction to vanadium causes a diffuse eczematous eruption and titanium alloy orthopedic implant failure. Cutis.

[28] Sinha S., Begam H., Kumar V., Nandi S.K., Kubacki J., Chanda A. (2018). Improved performance of the functionalized nitinol as a prospective bone implant material. J. Mater. Res..

[29] Jenko M., Godec M., Kocijan A., Rudolf R., Dolinar D., Ovsenik M., Gorenšek M., Mozetic M. (2019). A new route to biocompatible Nitinol based on a rapid treatment with H 2 /O 2 gaseous plasma. Appl. Surf. Sci..

[30] Shabalovskaya S., Anderegg J., Van Humbeeck J. (2008). Critical overview of Nitinol surfaces and their modifications for medical applications. Acta Biomater..

[31] Rubitschek F., Niendorf T., Karaman I., Maier H.J. (2012). Corrosion fatigue behavior of a biocompatible ultrafine-grained niobium alloy in simulated body fluid. J. Mech. Behav. Biomed. Mater..

[32] Pawar V., Weaver C., Jani S. (2011). Physical characterization of a new composition of oxidized zirconium-2.5 wt% niobium produced using a two step process for biomedical applications. Appl. Surf. Sci..

[33] Miyazaki S., Kim H.Y. (2011). Basic Characteristics of Titanium–Nickel (Ti–Ni)-Based and Titanium–Niobium (Ti–Nb)-Based Alloys.

[34] Kayhan S.M., Tahmasebifar A., Koç M., Usta Y., Tezcaner A., Evis Z. (2016). Experimental and numerical investigations for mechanical and microstructural characterization of micro-manufactured AZ91D magnesium alloy disks for biomedical applications. Mater. Des..

[35] Gu X.N., Zhou W.R., Zheng Y.F., Cheng Y., Wei S.C., Zhong S.P., Xi T.F., Chen L.J. (2010). Corrosion fatigue behaviors of two biomedical Mg alloys—AZ91D and WE43—In simulated body fluid. Acta Biomater..

[36] Lehr I.L., Saidman S.B. (2018). Corrosion protection of AZ91D magnesium alloy by a cerium-molybdenum coating. The effect of citric acid as an additive. J. Magnes. Alloy..

[37] Oonishi H., Kushitani S., Yasukawa E., Iwaki H., Hench L.L., Wilson J., Tsuji E., Sugihara T. (1997). Particulate bioglass compared with hydroxyapatite as a bone graft substitute. Clin. Orthop. Relat. Res..

[38] Descamps M., Richart O., Hardouin P., Hornez J.C., Leriche A. (2008). Synthesis of macroporous b -tricalcium phosphate with controlled porous architectural. Ceram. Int..

[39] Seebach C., Schultheiss J., Wilhelm K., Frank J., Henrich D. (2010). Comparison of six bone-graft substitutes regarding to cell seeding efficiency, metabolism and growth behaviour of human mesenchymal stem cells (MSC) In Vitro. Injury.

[40] Ducheyne P. (1985). Bioglass coatings and bioglass composites as implant materials. J. Biomed. Mater. Res..

[41] Hench L.L. (2006). The story of Bioglass. Mater. Med..

[42] Fabbri P., Cannillo V., Sola A., Dorigato A., Chiellini F. (2010). Highly porous polycaprolactone-45S5 Bioglass Ò scaffolds for bone tissue engineering. Compos. Sci. Technol..

[43] Aina V., Malavasi G., Fiorio Pla A., Munaron L., Morterra C. (2009). Zinc-containing bioactive glasses: Surface reactivity and behaviour towards endothelial cells. Acta Biomater..

[44] Krishnan V., Lakshmi T. (2013). Bioglass: A novel biocompatible innovation. J. Adv. Pharm. Technol. Res..

[45] Siriphannon P., Kameshima Y., Yasumori A. (2002). Formation of hydroxyapatite on CaSiO 3 powders in simulated body fluid. J. Eur. Ceram. Soc..

[46] De Aza P.N., Luklinska Z.B., Anseau M.R., Hector M., Guitia F., De Aza S. (2000). Reactivity of a wollastonite tricalcium phosphate Bioeutectic ceramic in human parotid saliva. Biomaterials.

[47] Xu S., Lin K., Wang Z., Chang J., Wang L., Lu J., Ning C. (2008). Reconstruction of calvarial defect of rabbits using porous calcium silicate bioactive ceramics. Biomaterials.

[48] Bratton E.M., Durairaj V.D. (2011). Orbital implants for fracture repair. Curr. Opin. Ophthalmol..

[49] Gomez d’Ayala G., De Rosa A., Laurienzo P., Malinconico M. (2006). Elastin Blends for Tissue Engineering Scaffolds. J. Biomed. Mater. Res. Part A.

[50] Vlad M.D., Şindilar E.V., Mariñoso M.L., Poeată I., Torres R., López J., Barracó M., Fernández E. (2010). Osteogenic biphasic calcium sulphate dihydrate/iron-modified α-tricalcium phosphate bone cement for spinal applications: In vivo study. Acta Biomater..

[51] Yashavantha Kumar C., Nalini K.B., Menon J., Patro D.K., Banerji B.H. (2013). Calcium sulfate as bone graft substitute in the treatment of osseous bone defects, a prospective study. J. Clin. Diagn. Res..

[52] Wahl P., Livio F., Jacobi M., Gautier E., Buclin T. (2011). Systemic exposure to tobramycin after local antibiotic treatment with calcium sulphate as carrier material. Arch. Orthop. Trauma Surg..

[53] Biradar S., Ravichandran P., Gopikrishnan R., Goornavar V., Hall J.C., Ramesh V., Baluchamy S., Jeffers R.B., Govindarajan R.T. (2011). Calcium carbonate nanoparticles: Synthesis, characterization and biocompatibility. J. Nanosci. Nanotechnol..

[54] Dizaj S.M., Barzegar-Jalali M., Hossein Zarrintan M., Adibkia K., Lotfipour F. (2015). Calcium carbonate nanoparticles; Potential in bone and tooth disorders. Pharm. Sci..

[55] Wang C., Chen S., Yu Q., Hu F., Yuan H. (2017). Taking advantage of the disadvantage: Employing the high aqueous instability of amorphous calcium carbonate to realize burst drug release within cancer cells. J. Mater. Chem. B.

[56] Almeida J.C., Wacha A., Gomes P.S., Fernandes M.H.R., Fernandes M.H.V., Salvado I.M.M. (2016). PDMS-SiO2-TiO2-CaO hybrid materials—Cytocompatibility and nanoscale surface features. Mater. Sci. Eng. C.

[57] Wu Y., Zitelli J.P., TenHuisen K.S., Yu X., Libera M.R. (2011). Differential response of Staphylococci and osteoblasts to varying titanium surface roughness. Biomaterials.

[58] Ma X., Feng Y., Ma Z., Li X., Wang J., Wang L. (2014). Biomaterials The promotion of osteointegration under diabetic conditions using chitosan/hydroxyapatite composite coating on porous titanium surfaces. Biomaterials.

[59] Carano R.A.D.D., Filvaroff E.H., Carano R.A.D.D., Filvaroff E.H. (2003). Angiogenesis and bone repair. Drug Discov. Today.

[60] Zardiackas L.D., Parsell D.E., Dillon L.D., Mitchell D.W., Nunnery L.A., Poggie R. (2001). Structure, metallurgy, and mechanical properties of a porous tantalum foam. J. Biomed. Mater. Res..

[61] Balla V.K., Bodhak S., Bose S., Bandyopadhyay A. (2010). Porous tantalum structures for bone implants: Fabrication, mechanical and in vitro biological properties. Acta Biomater..

[62] Karageorgiou V., Kaplan D. (2005). Porosity of 3D biomaterial scaffolds and osteogenesis. Biomaterials.

[63] Itl A.I., Ylnen H.O., Ekholm C., Karlsson K.H., Aro H.T. (2001). Pore diameter of more than 100 μm is not requisite for bone ingrowth in rabbits. J. Biomed. Mater. Res..

[64] Liu F.H. (2014). Fabrication of bioceramic bone scaffolds for tissue engineering. J. Mater. Eng. Perform..

[65] Deligianni D.D., Katsala N.D., Koutsoukos P.G., Missirlis Y.F. (2000). Effect of surface roughness of hydroxyapatite on human bone marrow cell adhesion, proliferation, differentiation and detachment strength. Biomaterials.

[66] Hench L.L. (1991). Bioceramics: From concept to clinic. J. Am. Ceram. Soc..

[67] Heakal F.E.T., Shehata O.S., Tantawy N.S. (2014). Integrity of metallic medical implants in physiological solutions. Int J. Electrochem. Sci..

[68] Koseki H., Tomita M., Yonekura A., Higuchi T., Sunagawa S., Baba K., Osaki M. (2017). Effect of carbon ion implantation on the tribology of metal-on-metal bearings for artificial joints. Int. J. Nanomed..

[69] Tavares D., Castro L., Soares G., Alves G., Granjeiro J. (2013). Synthesis and cytotoxicity evaluation of granular magnesium substituted β-tricalcium phosphate. J. Appl. Oral Sci..

[70] Himanshu T., Sp S., Ka S., Prerna M., Ashish J. (2016). Studies on preparation and characterization of 45S5 Bioactive glass doped with (TiO 2 + ZrO 2 ) as bioactive ceramic material. Bioceram. Dev. Appl..

[71] Arifin A., Sulong A.B., Muhamad N., Syarif J., Ramli M.I. (2014). Material processing of hydroxyapatite and titanium alloy (HA/Ti) composite as implant materials using powder metallurgy: A review. Mater. Des..

[72] Wang X., Li J., Hu R., Kou H., Zhou L. (2013). Mechanical properties of porous titanium with different distributions of pore size. Trans. Nonferr. Met. Soc. China.

[73] Nakai M., Niinomi M., Zhao X., Zhao X. (2011). Self-adjustment of Young’s modulus in biomedical titanium alloys during orthopaedic operation. Mater. Lett..

[74] Kuroda D., Niinomi M., Morinaga M., Kato Y., Yashiro T. (1998). Design and mechanical properties of new i type titanium alloys for implant materials. Mater. Sci. Eng. A.

[75] Keaveney S., Baron S., Ahearne E., Connolly P., Byrne G. An Assessment of Medical Grade Cobalt Chromium Alloy ASTM F1537 as a Difficult-to-Cut (DTC) Material An Assessment of Medical Grade Cobalt Chromium Alloy ASTM F1537 as a “Difficult-to-Cut (DTC)”; Material. https://www.researchgate.net/publication/287198713.

[76] Kato K., Yamamoto A., Ochiai S., Wada M., Daigo Y., Kita K., Omori K. (2013). Cytocompatibility and mechanical properties of novel porous 316 L stainless steel. Mater. Sci. Eng. C.

[77] Mi Z.R., Shuib S., Hassan A.Y., Shorki A.A., Ibrahim M.M. (2007). Problem of stress shielding and improvement to the hip implant designs: A Review. J. Med. Sci..

[78] Sumitomo N., Noritake K., Hattori T., Morikawa K., Niwa S., Sato K., Niinomi M. (2008). Experiment study on fracture fixation with low rigidity titanium alloy Plate fixation of tibia fracture model in rabbit. Mat. Med..

[79] Niinomi M., Liu Y., Nakai M., Liu H., Li H. (2016). Biomedical titanium alloys with Young’s moduli close to that of cortical bone. Regen. Biomater..

[80] Havaldar R., Pilli S., Putti B. (2014). Insights into the effects of tensile and compressive loadings on human femur bone. Adv. Biomed. Res..

[81] Yamada S., Tadano S., Fukuda S. (2014). Nanostructure and elastic modulus of single trabecula in bovine cancellous bone. J. Biomech..

[82] Cesar R., Leivas T.P., Pereira C.A.M., Boffa R.S., Guarniero R., de Menezes Reiff R.B., Netto A.M., Fortulan C.A., de Almeida Rollo J.M.D. (2017). Axial compressive strength of human vertebrae trabecular bones classified as normal, osteopenic and osteoporotic by quantitative ultrasonometry of calcaneus. Res. Biomed. Eng..

[83] Sanyal A., Gupta A., Bayraktar H.H., Kwon R.Y., Keaveny T.M. (2012). Shear strength behavior of human trabecular bone. J. Biomech..

[84] Gautam C.R., Kumar S., Biradar S., Jose S., Mishra V.K. (2016). Synthesis and enhanced mechanical properties of MgO substituted hydroxyapatite: A bone substitute material. RSC Adv..

[85] Mondal S., Pal U., Dey A. (2016). Natural origin hydroxyapatite scaffold as potential bone tissue engineering substitute. Ceram. Int..

[86] Liang L., Rulis P., Ching W.Y. (2010). Mechanical properties, electronic structure and bonding of α-and β-tricalcium phosphates with surface characterization. Acta Biomater..

[87] Tian Y., Lu T., He F., Xu Y., Shi H., Shi X., Zuo F., Wu S., Ye J. (2018). Β-Tricalcium phosphate composite ceramics with high compressive strength, enhanced osteogenesis and inhibited osteoclastic activities. Coll. Surf. B Biointerfaces.

[88] Shim J.H., Won J.Y., Park J.H., Bae J.H., Ahn G., Kim C.H., Lim D.H., Cho D.W., Yun W.S., Bae E.B. (2017). Effects of 3D-printed polycaprolactone/β-tricalcium phosphate membranes on guided bone regeneration. Int. J. Mol. Sci..

[89] Xie J., Yang X., Shao H., Juan Y., Yong H., Jianzhong F., Changyou G., Zhongru G. (2016). Simultaneous mechanical property and biodegradation improvement of wollastonite bioceramic through magnesium dilute doping. J. Mech. Behav. Biomed. Mater..

[90] Baino F., Pons E. (2019). Modelling the relationship between tensile strength and porosity in bioceramic scaffolds. Int. J. Appl. Ceram. Technol..

[91] Baino F., Fiume E. (2019). Elastic mechanical properties of 45S5-based bioactive glass-ceramic scaffolds. Materials.

[92] Řehořek L., Chlup Z., Meng D., Yunos D.M., Boccaccini A.R., Dlouhý I. (2013). Response of 45S5 Bioglass® foams to tensile loading. Ceram. Int..

[93] Wang Q., Eltit F., Wang R. (2018). Corrosion of Orthopedic Implants.

[94] Wakily H., Dabbagh A., Abdullah H., Abdul Halim N.F., Abu Kasim N.H. (2015). Improved thermal and mechanical properties in hydroxyapatite-titanium composites by incorporating silica-coated titanium. Mater. Lett..

[95] Wu S., Liu X., Gao C. (2015). Role of adsorbed proteins on hydroxyapatite-coated titanium in osteoblast adhesion and osteogenic differentiation. Sci. Bull..

[96] Lee H.-J., Kwon T.-Y., Kim K.-H., Kang S.S., Choi S.H., Kwon S.T., Cho D.H., Son J.S. (2015). In Vitro evaluation of hydroxyapatite-coated titanium implant with Atmospheric Plasma Treatment. J. Nanosci. Nanotechnol..

[97] Pillai R.S., Frasnelli M., Sglavo V.M. (2018). HA/β-TCP plasma sprayed coatings on Ti substrate for biomedical applications. Ceram. Int..

[98] Kumari R., Majumdar J.D. (2017). Microstructure and surface mechanical properties of plasma spray deposited and post spray heat treated hydroxyapatite (HA) based composite coating on titanium alloy (Ti-6Al-4V) substrate. Mater. Charact..

[99] Qiao S.C., Du J., Zhao J.M., Shi J.Y., Gu Y.X., Lai H.C. (2015). Effects of a hydroxyapatite-coated nanotube surface of titanium on MC3T3-E1 cells: An In Vitro study. Implant. Dent..

[100] Catauro M. (2017). Response In Vitro Human Cells to Hydroxyapatite Coatings on Titanium Substrates Synthesized by Sol-Gel Process. EC Dent. Sci..

[101] Yang Y., Kim K.H., Agrawal C.M., Ong J.L. (2004). Interaction of hydroxyapatite-titanium at elevated temperature in vacuum environment. Biomaterials.

[102] Tan X.W., Beuerman R.W., Shi Z.L., Neoh K.G., Tan D., Khor K.A., Mehta J.S. (2012). In Vivo evaluation of titanium oxide and hydroxyapatite as an artificial cornea skirt. J. Mater. Sci. Mater. Med..

[103] Costa De Almeida C., Sena L.Á., Pinto M., Muller C.A., Cavalcanti Lima J.H., Soares G.D.A. (2005). In Vivo characterization of titanium implants coated with synthetic hydroxyapatite by electrophoresis. Braz. Dent. J..

[104] Wang H., Eliaz N., Xiang Z., Hsu H.P., Spector M., Hobbs L.W. (2006). Early bone apposition in vivo on plasma-sprayed and electrochemically deposited hydroxyapatite coatings on titanium alloy. Biomaterials.

[105] Palakurthy S., Samudrala R.K. (2019). In vitro bioactivity and degradation behaviour of β-wollastonite derived from natural waste. Mater. Sci. Eng. C.

[106] Hossain S.S., Yadav S., Majumdar S., Krishnamurthy S., Pyare R., Roy P.K. (2020). A comparative study of physico-mechanical, bioactivity and hemolysis properties of pseudo-wollastonite and wollastonite glass-ceramic synthesized from solid wastes. Ceram. Int..

[107] Obeid M.M. (2014). Crystallization of synthetic wollastonite prepared from local raw materials. Int J. Mater. Chem..

[108] Rahmani R., Antonov M., Kollo L., Holovenko Y., Prashanth K.G. (2019). Mechanical behavior of Ti6Al4V scaffolds filled with CaSiO3 for implant applications. Appl. Sci..

[109] Aly I.H.M., Abed Alrahim Mohammed L., Al-Meer S., Elsaid K., Barakat N.A.M. (2016). Preparation and characterization of wollastonite/titanium oxide nanofiber bioceramic composite as a future implant material. Ceram. Int..

[110] Solonenko A.P., Blesman A.I., Polonyankin D.A. (2018). Preparation and in vitro apatite-forming ability of hydroxyapatite and β-wollastonite composite materials. Ceram. Int..

[111] Zhao X., Wang T., Qian S., Liu X., Sun J., Li B. (2016). Silicon-doped titanium dioxide nanotubes promoted bone formation on titanium implants. Int. J. Mol. Sci..

[112] Arora M., Arora E. (2017). The promise of silicon: Bone regeneration and increased bone density. J. Arthrosc. Jt. Surg..

[113] Sampath S., Herman H. (1996). Rapid solidification and microstructure development during plasma spray deposition. J. Therm. Spray Technol..

[114] Fu Q., Hong Y., Liu X., Fan H., Zhang X. (2011). A hierarchically graded bioactive scaffold bonded to titanium substrates for attachment to bone. Biomaterials.

[115] Flame Spray Technologies APS Plasma Thermal Spray Coating System. https://www.fst.nl/thermal-spray-equipment/modular-thermal-spray-systems/plasma-thermal-spray-systems.html.

[116] Levingstone T.J., Ardhaoui M., Benyounis K., Looney L., Stokes J.T. (2015). Plasma sprayed hydroxyapatite coatings: Understanding process relationships using design of experiment analysis. Surf. Coat. Technol..

[117] Arifin A., Sulong A.B., Muhamad N., Syarif J., Ramli M.I. (2015). Powder injection molding of HA/Ti6Al4V composite using palm stearin as based binder for implant material. Mater. Des..

[118] Ramli M.I., Sulong A.B., Muhamad N., Muctar A., Ng M.H., Shanmuganantha L. Ti6Al4V/Wollastonite composite through powder injection molding process for bone implant application. Proceedings of the International Medical Device and Technology Conference.

[119] Chen L., Li T., Li Y., He H., Hu Y. (2009). Porous titanium implants fabricated by metal injection molding. Trans. Nonferr. Met. Soc. China.

[120] Shivashankar T.S., German R.M. (1999). Effective length scale for predicting solvent-debinding times of components produced by powder injection molding. J. Am. Ceram. Soc..

[121] Ye H., Yang X., Hong H. (2007). Fabrication of metal matrix composites by metal injection molding—A review. J. Mater. Process. Technol..

[122] Todd R.H., Allen D.K., Alting L. (2019). Manufacturing Processes Reference Guide.

[123] Abida F., Elassfouri M., Ilou M., El B., Jamil M., Moncif N. (2017). Tricalcium phosphate powder: Preparation, characterization and compaction abilities. Mediterr. J. Chem..

[124] Rodzi S.N.H.M., Zuhailawati H. (2016). The effects of processing techniques on magnesium-based composite. AIP Conf. Proc..

[125] Ragurajan D., Satgunam M., Golieskardi M., Sankar U., Ng A.M.H. (2019). The effect of titanium oxide and hydroxyapatite on the mechanical properties of wollastonite. Cogent. Eng..

[126] Chu C., Xue X., Zhu J., Yin Z. (2006). Fabrication and characterization of titanium-matrix composite with 20 vol% hydroxyapatite for use as heavy load-bearing hard tissue replacement. J. Mater. Sci. Mater. Med..

[127] Peñarrieta-Juanito G.M., Costa M., Cruz M., Miranda G., Henriques B., Marques J., Magini R., Mata A., Caramês J., Silva F. (2018). Bioactivity of novel functionally structured titanium-ceramic composites in contact with human osteoblasts. J. Biomed. Mater. Res. Part. A.

[128] Limongi T., Tirinato L., Pagliari F., Giugni A., Allione M., Perozziello G., Candeloro P., Di Fabrizio E. (2017). Fabrication and applications of micro/nanostructured devices for tissue engineering. Nano Micro Lett..

[129] Bose S., Ke D., Sahasrabudhe H., Bandyopadhyay A. (2018). Progress in materials science additive manufacturing of biomaterials. Prog. Mater. Sci..

[130] Campoli G., Borleffs M.S., Amin Yavari S., Wauthle R., Weinans H., Zadpoor A.A. (2013). Mechanical properties of open-cell metallic biomaterials manufactured using additive manufacturing. Mater. Des..

[131] Melancon D., Bagheri Z.S., Johnston R.B., Liu L., Tanzer M., Pasini D. (2017). Mechanical characterization of structurally porous biomaterials built via additive manufacturing: Experiments, predictive models, and design maps for load-bearing bone replacement implants. Acta Biomater..

[132] Vlad M.D., Fernández Aguado E., Gómez González S., Ivanov I.C., Şindilar E.V., Poeată I., Iencean A.Ş., Butnaru M., Avădănei E.R., López J.L. (2020). Novel titanium-apatite hybrid scaffolds with spongy bone-like micro architecture intended for spinal application: In vitro and in vivo study. Mater. Sci. Eng. C.

[133] Jaiswal S., Kumar R.M., Gupta P., Kumaraswamy M., Roy P., Lahiri D. (2017). Mechanical, corrosion and biocompatibility behaviour of Mg-3Zn-HA biodegradable composites for orthopaedic fixture accessories. J. Mech. Behav. Biomed. Mater..

[134] Wang Z.L., He R.Z., Tu B., Cao X., He J.S., Xia H.S., Liang C., Zou M., Wu S., Wu Z.J. (2017). Enhanced biocompatibility and osseointegration of calcium titanate coating on titanium screws in rabbit femur. J. Huazhong Univ. Sci. Technol. Med. Sci..

[135] Lukaszewska-Kuska M., Wirstlein P., Majchrowski R., Dorocka-Bobkowska B. (2018). Osteoblastic cell behaviour on modified titanium surfaces. Micron.

[136] Spoerke E.D., Murray N.G., Li H., Brinson L.C., Dunand D.C., Stupp S.I. (2005). A bioactive titanium foam scaffold for bone repair. Acta Biomater..

[137] Kohn D.H., Sarmadi M., Helman J.I., Krebsbach P.H. (2001). Effects of pH on human bone marrow stromal cells in vitro: Implications for tissue engineering of bone. J. Biomed. Mater. Res. Off. J. Soc. Biomater. Jpn. Soc. Biomater. Aust. Soc. Biomater. Korean Soc. Biomater..

[138] Bergsma J.E., Bruijn WCDe Rozema F.R., Bos R.R.M., Boering G. (1995). Late degradation tissue response to poly ( L-lactide ) bone plates and screws. Biomaterials.

[139] Li J. (1993). Behaviour of titanium and titania-based ceramics In Vitro and In Vivo. Biomaterials.

[140] Fartash B., Liao H., Li J., Fouda N., Hermansson L. (1995). Long-term evaluation of titania-based ceramics compared with commercially pure titanium in vivo. J. Mater. Sci. Mater. Med..

[141] Road P., Sachlos E., Czernuszka J.T. (2003). Making tissue engineering scaffolds work. Review on the application of solid freeform fabrication technology to the production of tissue engineering scaffolds. Eur. Cell Mater..

[142] Manivasagam G., Dhinasekaran D., Rajamanickam A. (2010). Biomedical Implants: Corrosion and its Prevention—A Review. Recent Pat. Corosion Sci..

[143] Oldani C., Dominguez A., Eli T. (2012). Titanium as a biomaterial for implants. Recent Adv. Arthroplast..

[144] Stryker AlloCraft CA Cervical Allograft Spacer. https://www.stryker.com/us/en/spine/products/allocraft-ca.html.

[145] Stryker BIO Wedge. https://www.stryker.com/us/en/spine/products/bio-wedge.html.

[146] Stryker ReUnion RSA. https://www.stryker.com/us/en/trauma-and-extremities/products/reunion-rsa.html.

[147] Stryker ReUnion S Humeral Stem. https://www.stryker.com/us/en/trauma-and-extremities/products/ReUnion-S.html.

[148] Stryker T2 Humeral Fractures Humeral Nailing System. https://www.stryker.com/us/en/trauma-and-extremities/products/t2-standard-humeral-nail.html.

[149] Zimmer Biomet OsseoTi ® Porous Metal Technology. https://www.zimmerbiomet.com/medical-professionals/common/our-science/osseoti-porous-metal.html.

[150] Zimmer Biomet Versa-Fx ® II Femoral Fixation. https://www.zimmerbiomet.com/medical-professionals/trauma/product/versa-fx-ii-femoral-fixation-system.html.

[151] Zimmer Biomet NexGen ® Legacy ® Constrained Condylar Knee ( LCCK ) Tibial and Femoral Augments. https://www.zimmerbiomet.com/medical-professionals/knee/product/nexgen-lcck.html.

[152] Zimmer Biomet Avenir ® Hip System Avenir Cementless Stem Surface Finish and Macro Structure Meant To Last. https://www.zimmerbiomet.com/content/dam/zimmer-biomet/medical-professionals/hip/avenir-hip-system/0725.1-GLBL-en%20Avenir%20Hip%20System_Brochure_FINAL.pdf.

[153] TrellOss ^TM^ -C Porous Ti Interbody System. https://www.zimmerbiomet.com/medical-professionals/spine/product/trelloss-ts-porous-ti-interbody-system.html.

[154] DePuy Synthes ACTIS® Total Hip System. https://www.jnjmedicaldevices.com/en-EMEA/product/actis-total-hip-system.

[155] DePuy Synthes ATTUNE ® Revision Knee System. https://www.jnjmedicaldevices.com/en-US/product/attune-revision-knee-system.

[156] DePuy Synthes GLOBAL ® CAP Conservative Anatomic Prosthesis & GLOBAL ® CAP CTA ^TM^ Cu Tear Arthropathy Conservative Prosthesis Features. https://www.jnjmedicaldevices.com/en-US/product/globalr-cap-conservative-anatomic-prosthesis-globalr-cap-ctatm-cuff-tear-arthropathy.

[157] DePuy Synthes SIGMA ® Total Knee System. https://www.jnjmedicaldevices.com/en-US/product/sigmar-total-knee-system.

[158] DePuy Synthes PROTI 360° ^TM^ Ti Integrated Technology. https://www.jnjmedicaldevices.com/en-US/product/proti-360degtm-ti-integrated-technology.

[159] Smith and Nephew Revision Hip System. https://www.smith-nephew.com/key-products/orthopaedic-reconstruction/redapt/.

[160] Smith and Nephew VERILAST ◊ Technology for Hips. https://www.smith-nephew.com/professional/products/all-products/verilast-technology/.

[161] Smith and Nephew POLAR3 ◊. https://www.smith-nephew.com/professional/products/all-products/polar3/.

[162] Smith and Nephew BIOSURE ◊ HA and PK. https://www.smith-nephew.com/new-zealand/advanced-surgical-devices/key-products/sports-medicine/biosure-ha-and-biosure-pk-interference-screws/.

[163] Smith and Nephew CARGEL ◊ Bioscaffold Proven Performance. https://www.smith-nephew.com/key-products/sports-medicine/bst-cargel/.

[164] Elbuluk A.M., Old A.B., Bosco J.A., Schwarzkopf R., Iorio R. (2017). Strategies for reducing implant costs in the revision total knee arthroplasty episode of care. Arthroplast. Today.

[165] Parikh H.R., O’Hara N., Levy J.F., Cunningham B.P. (2019). Value denominator: The fundamentals of costing for orthopaedic surgeons. J. Orthop. Trauma.

[166] Arliani G.G., Sabongi R.G., Batista A.F., Astur D.C., Falotico G.G., Cohen M. (2016). Evaluation of the knowledge on cost of orthopedic implants among orthopedic surgeons. Acta Ortop. Bras..

[167] Xi’an Lyphar Biotech Co. Ltd. Lyphar Provide Most CAS NO 1306-06-5 Competitive Lyphar Provide Most CAS NO 1306-06-5 Competitive. https://www.cphi-online.com/xian-lyphar-biotech-co-ltd-comp266352.html.

[168] Xi’an Sgonek Biological Technology Co. Ltd. ISO Certificated Factory Supply CAS 1306-06-5 Calcium Hydroxyapatite Powder Hydroxyapatite. https://sgonekbio.en.alibaba.com/.

[169] Shanghai Ximeng New Materials Technology Co. Ltd. Hydroxyapatite powder CAS. https://cnshximeng.en.alibaba.com/.

[170] Richin International Trade (Dalian) Co. Ltd. Wollastonite Pictures of Wollastonite Product Description of Wollastonite. http://www.richase.com/.

[171] Shanghai CNPC Powder Material Co. Ltd. Low Price Factory Supply Wollastonite Powder Price. https://cnpcpowder.en.made-in-china.com/.

[172] Shenyang Huakuang Trading Co. Ltd. Wollastonite Provider. http://www.syhkmining.com/contactus.

[173] Wilson N.A., Schneller E.S., Montgomery K., Bozic K.J. (2008). Hip and knee implants: Current trends and policy considerations. Health Aff..

